# PSGL-1 on Leukocytes is a Critical Component of the Host Immune Response against Invasive Pneumococcal Disease

**DOI:** 10.1371/journal.ppat.1005500

**Published:** 2016-03-14

**Authors:** Elisa Ramos-Sevillano, Ana Urzainqui, Belén de Andrés, Rafael González-Tajuelo, Mirian Domenech, Fernando González-Camacho, Francisco Sánchez-Madrid, Jeremy S. Brown, Ernesto García, Jose Yuste

**Affiliations:** 1 Centro de Investigaciones Biológicas (CIB-CSIC), Madrid, Spain; 2 CIBER de Enfermedades Respiratorias (CIBERES), Madrid, Spain; 3 Centro Nacional de Microbiología, Instituto de Salud Carlos III (ISCIII), Madrid, Spain; 4 Department of Immunology, Instituto Investigación Sanitaria Princesa (IIS-IP), Hospital de la Princesa, Madrid, Spain; 5 Centre for Inflammation and Tissue Repair, University College London, London, United Kingdom; University of Birmingham, UNITED KINGDOM

## Abstract

Bacterial uptake by phagocytic cells is a vital event in the clearance of invading pathogens such as *Streptococcus pneumoniae*. A major role of the P-selectin glycoprotein ligand-1 (PSGL-1) on leukocytes against invasive pneumococcal disease is described in this study. Phagocytosis experiments using different serotypes demonstrated that PSGL-1 is involved in the recognition, uptake and killing of *S*. *pneumoniae*. Co-localization of several clinical isolates of *S*. *pneumoniae* with PSGL-1 was demonstrated, observing a rapid and active phagocytosis in the presence of PSGL-1. Furthermore, the pneumococcal capsular polysaccharide and the main autolysin of the bacterium ―the amidase LytA― were identified as bacterial ligands for PSGL-1. Experimental models of pneumococcal disease including invasive pneumonia and systemic infection showed that bacterial levels were markedly increased in the blood of *PSGL-1* ^−/−^ mice. During pneumonia, PSGL-1 controls the severity of pneumococcal dissemination from the lung to the bloodstream. In systemic infection, a major role of PSGL-1 in host defense is to clear the bacteria in the systemic circulation controlling bacterial replication. These results confirmed the importance of this receptor in the recognition and clearance of *S*. *pneumoniae* during invasive pneumococcal disease. Histological and cellular analysis demonstrated that *PSGL-1* ^−/−^ mice have increased levels of T cells migrating to the lung than the corresponding wild-type mice. In contrast, during systemic infection, *PSGL-1* ^−/−^ mice had increased numbers of neutrophils and macrophages in blood, but were less effective controlling the infection process due to the lack of this functional receptor. Overall, this study demonstrates that PSGL-1 is a novel receptor for *S*. *pneumoniae* that contributes to protection against invasive pneumococcal disease.

## Author Summary

*S*. *pneumoniae* is one of the most important and devastating human pathogens worldwide, mainly affecting young children, elderly people and immunocompromised patients. In terms of host immune defense against invasive pneumococcal isolates, professional phagocytes require receptor-mediated recognition of certain ligands on the bacterial surface for the uptake and clearance of the microorganism. In this study, we demonstrate that the P-selectin glycoprotein ligand-1 (PSGL-1) on leukocytes is involved in the phagocytosis process of *S*. *pneumoniae* by targeting the capsule and the surface protein LytA as pathogen-associated molecular patterns. To explore this process in more detail, we have used wild-type mice and mice deficient in PSGL-1 demonstrating that lack of PSGL-1 is detrimental for the host by increasing the susceptibility to the infection and the severity of the pneumococcal invasive disease. Overall, these data show the importance of PSGL-1 on leukocytes in host defense against *S*. *pneumoniae* and confirm that PSGL-1 plays a critical protective role against invasive bacterial disease.

## Introduction

*Streptococcus pneumoniae* (pneumococcus) is one of the major causes of invasive disease accounting for more deaths than any other vaccine-preventable bacterial infection. This microorganism colonizes the human nasopharynx, being one of the leading causes of acute otitis media, community-acquired pneumonia and invasive pneumococcal disease (IPD) including sepsis and meningitis [1]. The World Health Organization estimates that nearly 14 million episodes of serious pneumococcal disease occur every year with a critical impact in childhood population as pneumonia kills more children than AIDS, malaria and measles combined [1, 2].

Resolution of pneumococcal disease is regulated by the efficient recognition and clearance of the invading pathogen by professional phagocytes [3]. Leukocytes play an important role in inflammatory and immune responses against bacterial infection and bacterial clearance depends on the efficacy of different receptors on phagocytic cells to recognize, internalize and kill the pathogen [4–7]. Phagocytic receptors on the cell surface trigger phagocytosis following direct recognition of particulate targets. Interaction between selectins and selectin-ligand molecules is essential for the host-pathogen encounter due to its crucial role in leukocyte extravasation [8]. In this sense, expression of P-selectin and E-selectin by the endothelium provides protection against invading pathogens such as *S*. *pneumoniae* [9, 10]. P-selectin glycoprotein ligand-1 (PSGL-1) on leukocytes mediates interactions with P-selectin and E-selectin expressed by endothelial cells [11]. PSGL-1 is a homodimeric mucin-like glycoprotein expressed on the surface of almost all circulating leukocytes with a great importance in leukocyte adhesion and transmigration as it is responsible for the initial steps of the extravasation cascade [8, 12]. However, certain intracellular pathogens have developed sophisticated strategies exploiting specific receptors for their own benefit to enter eukaryotic cells and replicate intracellularly [13]. This is the case of the obliged intracellular pathogens *Anaplasma phagocytophilum*, *Ehrlichia* sp., and enterovirus 71 that get access inside the cell by binding PSGL-1, causing granulocytic anaplasmosis/ehrlichiosis and hand-foot-mouth disease respectively [14–16]. However, there is no experimental evidence indicating that PSGL-1 could act as a receptor on leukocytes participating in the recognition and clearance of extracellular invading pathogens such as *S*. *pneumoniae*. In this sense, the main goal of this study was to investigate the protective contribution of PSGL-1 in host defense against IPD.

## Results

### PSGL-1 is a functional receptor in neutrophils involved in the phagocytic process of *S*. *pneumoniae*

**Fig 1:**
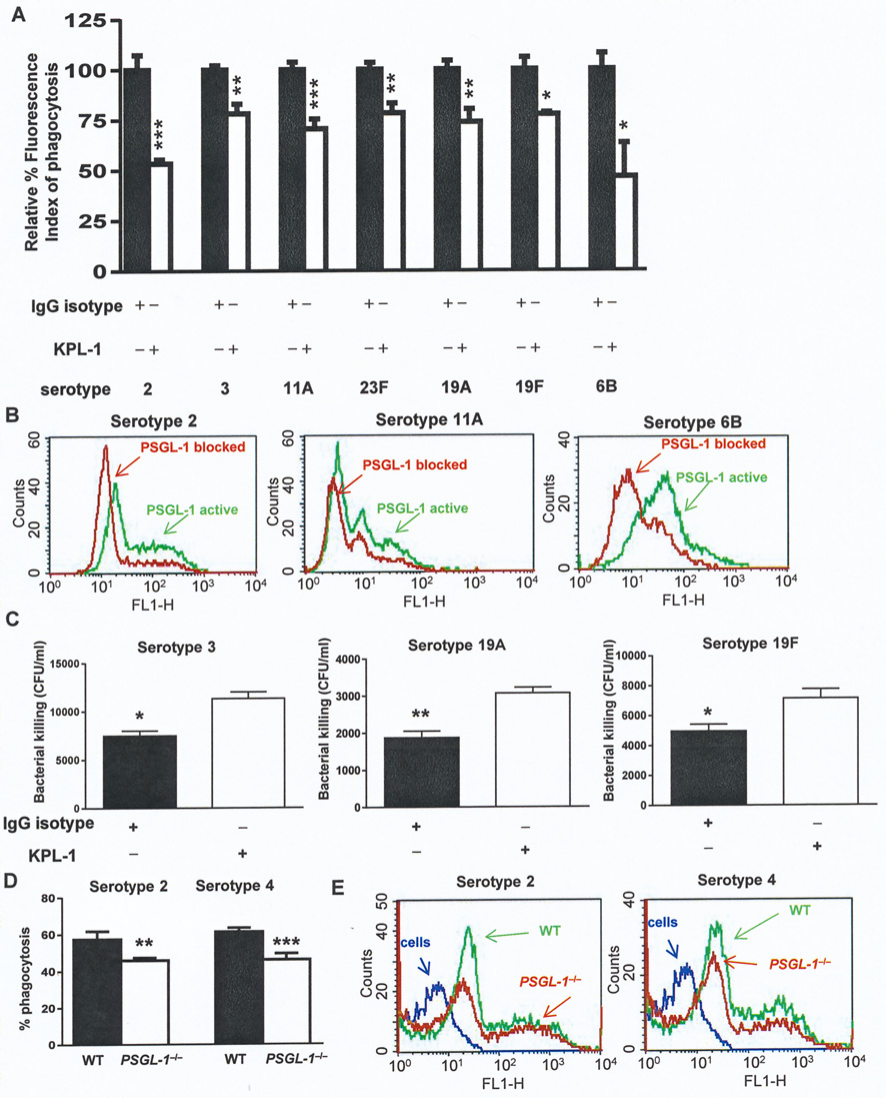
PSGL-1 on HL-60 cells and murine neutrophils is required for phagocytosis of *S*. *pneumoniae*. (A) Phagocytosis mediated by PSGL-1 of FAM-SE-labeled pneumococcal clinical isolates belonging to different serotypes using a flow cytometry assay. HL-60 cells were incubated with an IgG isotype negative control that does not block PSGL-1 (black bars), whereas cells exposed to the KPL-1 antibody had the PSGL-1 blocked (open bars). (B) Examples of flow cytometry histograms of pneumococcal binding to cells incubated with KPL-1 (red) or IgG control (green). (C) Bacterial survival indicated as CFU/ml of recovered bacteria from HL-60 cells previously exposed (open bars) or not (black bars) to KPL-1. (D−E) Phagocytosis of FAM-SE-labeled D39 strain by neutrophils isolated from the spleen of wild-type (black bar and green histogram) or *PSGL-1* ^–/–^mice (open bar and red histogram). The blue line in the histogram shows the pattern of non-infected cells. Error bars represent the SDs and asterisks indicate statistical significance after incubation with KPL-1 compared to the exposure to IgG isotype control or between OP using neutrophils from wild-type mice *vs* those from *PSGL-1* ^–/–^mice. Error bars represent the SDs and asterisks indicate statistical significance after incubation with KPL-1 compared to the exposure to IgG isotype control.

The plasma membrane of phagocytes expresses an array of receptors that interact with specific microbial ligands promoting the internalization and clearance of the potential pathogen. To evaluate the impact of PSGL-1 in pneumococcal phagocytosis, HL-60 cells differentiated to granulocytes were used as target cells because they express the same phagocytic receptors as peripheral blood neutrophils including PSGL-1 [14–17] (S1 Fig). To assess the role of PSGL-1 in the phagocytosis of *S*. *pneumoniae*, the receptor function was blocked using the specific monoclonal antibody KPL-1. This is an accepted method to assess the impact of PSGL-1 in microbial interaction [14–16]. To determine the generic role of PSGL-1 in host defense against this important pathogen, clinical isolates of *S*. *pneumoniae* belonging to different serotypes were assessed. Phagocytosis was significantly impaired when PSGL-1 was blocked, indicating that pneumococcal phagocytosis is more efficient when this receptor is fully active (Fig 1A and 1B). The contribution of FCγ-receptors was evaluated indicating that the effect of PSGL-1 in phagocytosis is independent of FCγ-receptors activity (S1 Fig). In addition, bacterial killing mediated by PSGL-1 was examined using three different clinical isolates. Our results showed that pneumococcal survival increased when PSGL-1 in phagocytic cells was blocked, demonstrating that this receptor is involved in the clearance of *S*. *pneumoniae* (Fig 1C). Finally, phagocytosis experiments using neutrophils obtained from the spleen of wild-type and *PSGL-1* ^–/–^mice by cell sorting, confirmed that PSGL-1 is involved in the phagocytosis of *S*. *pneumoniae* (Fig 1D and 1E).

**Fig 2:**
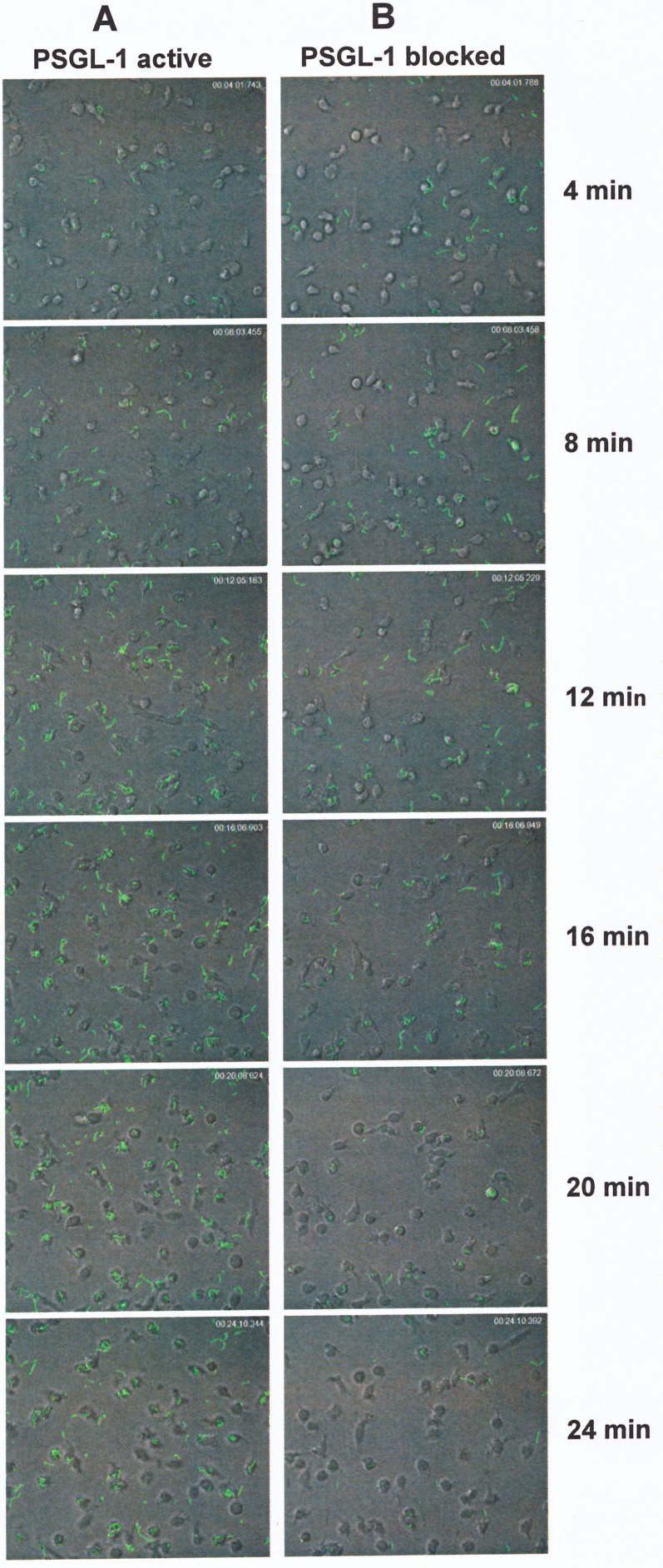
PSGL-1 contributes to the uptake of *S*. *pneumoniae*. Kinetics of pneumococcal phagocytosis of *S*. *pneumoniae* D39 (serotype 2) strain expressing GFP mediated by PSGL-1. Images are visualized at different times by live imaging confocal microscopy and environmental control (A) Phagocytosis of D39 strain using HL-60 cells with the PSGL-1 active. (B) Phagocytosis of D39 strain by HL-60 cells with the PSGL-1 blocked after incubation with KPL-1.

**Fig 3:**
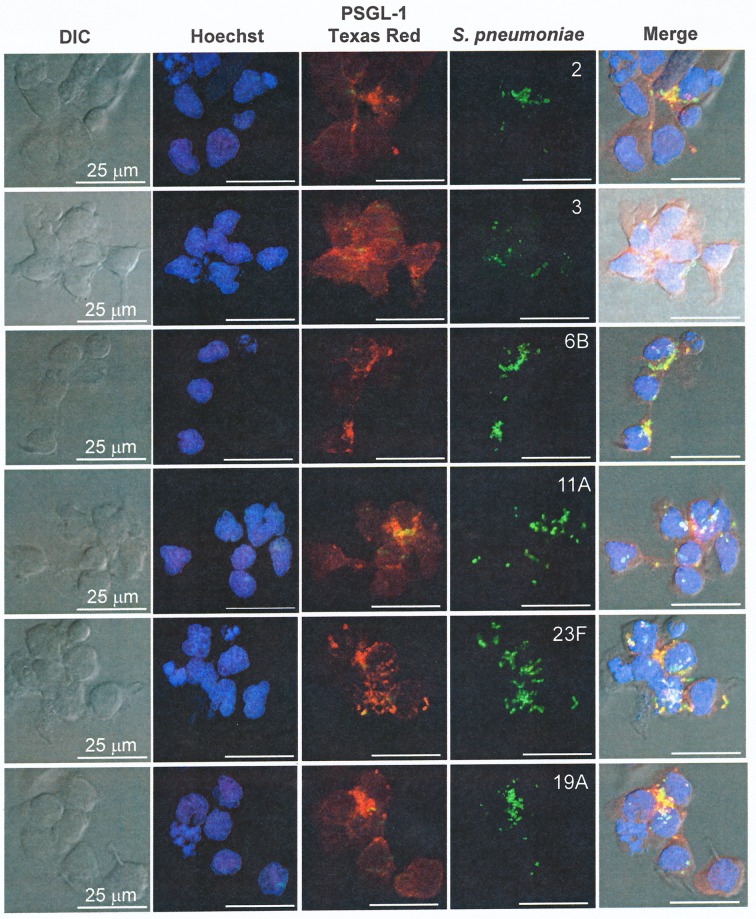
Recognition of *S*. *pneumoniae* by PSGL-1 is a generic event. Co-localization of FAMS-SE-labeled *S*. *pneumoniae* clinical isolates of different serotypes with PSGL-1 in HL-60 cells differentiated to neutrophils. Cellular DNA was stained with Hoechst whereas PSGL-1 was visualized with mouse anti-human PSGL-1 antibody followed by a goat anti-mouse Texas-Red staining. (DIC, Differential interference contrast).

To further analyze the kinetics of pneumococcal phagocytosis, cells with PSGL-1 ―either active or antibody-blocked― were infected with the D39 (serotype 2) strain expressing the green fluorescent protein (GFP), and the phagocytosis process was monitored using live imaging confocal microscopy (Fig 2A and 2B, S1 and S2 Movies). When PSGL-1 was active, a rapid and active phagocytosis was observed, with the majority of the cells containing pneumococcal cells by the end of the process (Fig 2A and S1 Movie). However, when PSGL-1 receptor was blocked, the recognition and engulfment of *S*. *pneumoniae* was impaired, which confirmed the importance of PSGL-1 in pneumococcal phagocytosis (Fig 2B and S2 Movie). To confirm the interaction of *S*. *pneumoniae* with PSGL-1, fluorescently-labeled pneumococcal isolates of serotypes 2, 3, 6B, 11A, 23F and 19A were used to observe co-localization with PSGL-1 (Fig 3 and S2 Fig). Hence, our findings show that PSGL-1 is involved in the recognition and phagocytosis of a major human pathogen such as *S*. *pneumoniae* contributing therefore, to the variety of receptors on professional phagocytes that are needed to efficiently identify and destroy invading pathogens [4–6, 18].

### Autolysin LytA and pneumococcal capsular polysaccharide are bacterial ligands recognized by PSGL-1

**Fig 4:**
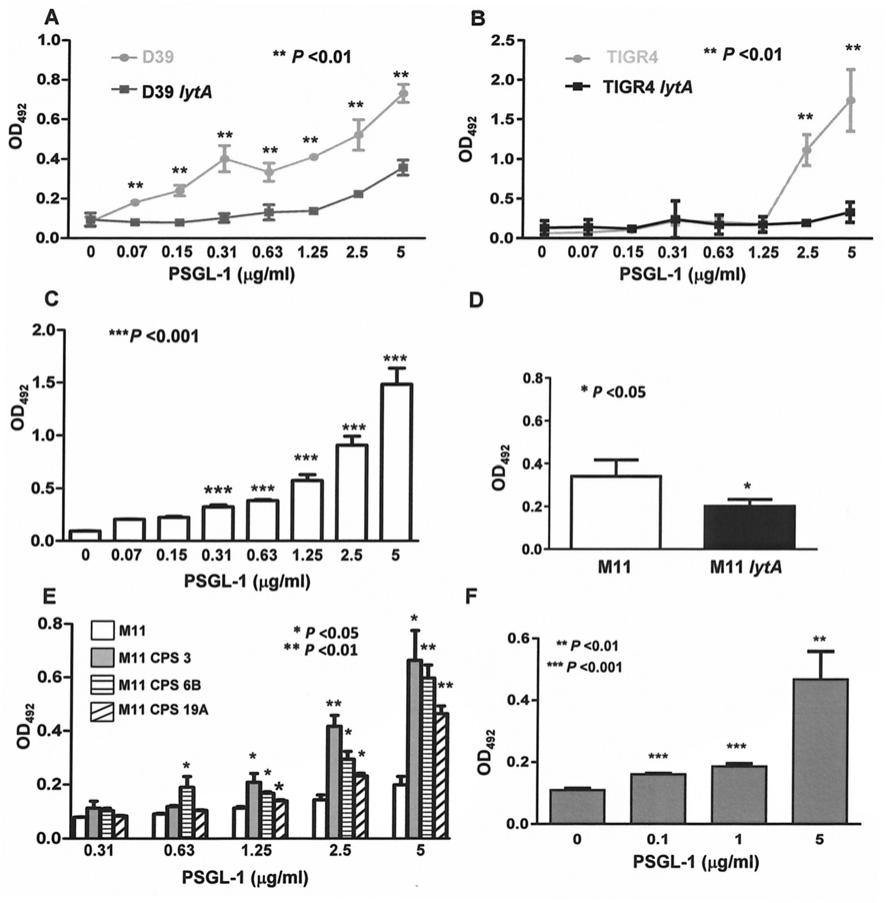
PSGL-1 recognizes the pneumococcal LytA autolysin and the capsular polysaccharide. (A) ELISA showing binding of different concentrations of PSGL-1 to *S*. *pneumoniae* D39 (gray circles) and its isogenic *lytA* mutant (black squares). (B) ELISA showing binding of different concentrations of PSGL-1 to *S*. *pneumoniae* TIGR4 (gray circles) and its isogenic *lytA* mutant (black squares). (C) ELISA representing binding of 50 μg of purified LytA protein to different concentrations of PSGL-1. (D) ELISA representing binding of 0.5 μg/ml of PSGL-1 to M11 strain and an isogenic M11 *lytA* mutant. (E) ELISA showing binding of different concentrations of PSGL-1 to the unencapsulated M11 strain (open bars) and the corresponding isogenic transformants expressing CPS of serotypes 3 (gray bars), 6B (striped bars) and 19A (hatched bars). (F) ELISA indicating binding of 50 μg of CPS of serotype 3 (CPS3) to different concentrations of PSGL-1. Error bars represent the SDs and asterisks indicate significant *P*-values for comparison of results of *S*. *pneumoniae* wild-type strains versus the corresponding mutants. For the direct recognition between PSGL-1 and LytA or CPS, asterisks indicate statistical significance of purified LytA protein or CPS3 incubated with PSGL-1 in comparison to the incubation of LytA or CPS3 in the absence of PSGL-1

Phagocytosis requires receptor-mediated recognition of microbial ligands that are usually expressed in the surface of invading pathogens. These ligands are frequently known as pathogen-associated molecular patterns (PAMPS) which are recognized by specific receptors of the innate immune system [19]. As LytA ―the main cell wall hydrolase of *S*. *pneumoniae*― is located on the bacterial surface and it is essential to interact with critical components of the host immune response including neutrophils and macrophages [20, 21], we explored the possible interaction of PSGL-1 with LytA. Pneumococcal recognition by PSGL-1 was hindered in the absence of LytA, suggesting that LytA might be a bacterial ligand for PSGL-1 (Fig 4A and 4B). Direct interaction between purified LytA and PSGL-1 molecules was observed confirming that this receptor recognizes LytA (Fig 4C). This interaction was dependent on the concentration of PSGL-1, suggesting that recognition of *S*. *pneumoniae* is enhanced when PSGL-1 levels are increased. Preincubation of HL-60 cells with purified LytA before infection reduced the phagocytosis in a similar way than KPL-1 antibody, supporting additional evidence that LytA interacts with PSGL-1 (S3 Fig). To confirm the interaction of LytA and PSGL-1 we included the non-capsulated strain (M11) and an isogenic *lytA* mutant strain (Fig 4D). Our results demonstrated that in the absence of LytA, the binding of *S*. *pneumoniae* to PSGL-1 is impaired. Although the capsular polysaccharide (CPS) is one of the major virulence factors of *S*. *pneumoniae*, resistance to phagocytosis can vary with the capsular type, which might explain differences of invasiveness among strains [22, 23]. Capsule recognition by PSGL-1 was investigated using a non-capsulated strain (M11) and several isogenic transformants of M11 expressing different CPSs (Fig 4E). The absence of CPS caused impaired recognition by PSGL-1, in comparison to the corresponding encapsulated transformants, confirming that PSGL-1 recognizes the pneumococcal CPS (Fig 4E). However, different levels of binding were observed depending on the CPS with the weakest recognition for the pneumococcal strain expressing serotype 19A (Fig 4E). Experiments including purified CPS of type 3 and different concentrations of PSGL-1 were included to investigate the binding of PSGL-1 to the pneumococcal capsule (Fig 4F). This CPS was assessed because is included in the current PCV-13 vaccine and clinical isolates of serotype 3 are a major cause of IPD [24]. Direct recognition of purified CPS by PSGL-1 was observed showing a concentration-dependent pattern, which confirms that PSGL-1 recognizes the capsule of *S*. *pneumoniae* (Fig 4F).

### PSGL-1 on leukocytes controls the severity of pneumococcal infection

**Fig 5:**
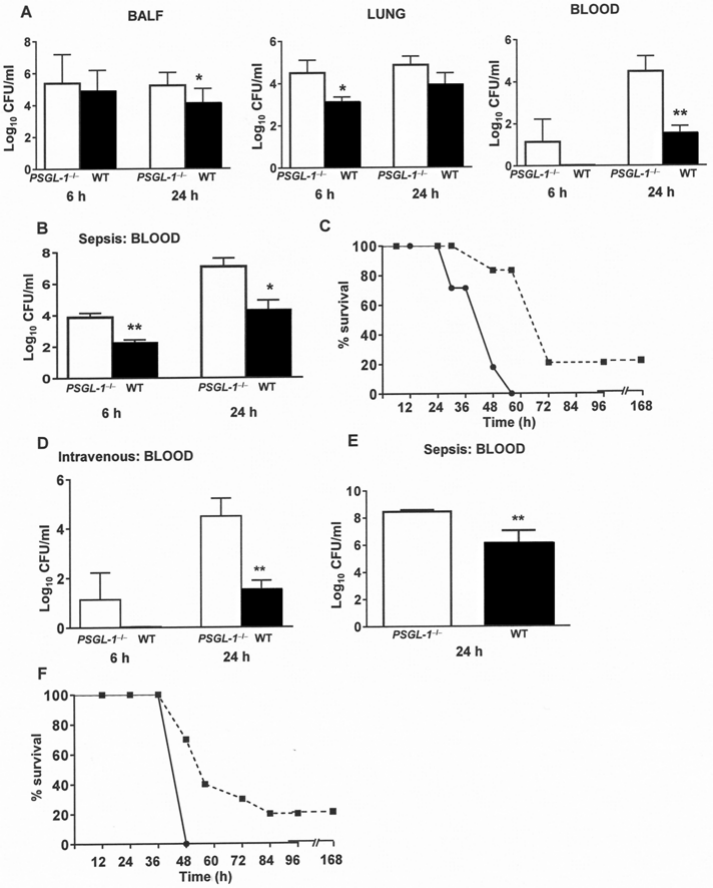
Contribution of PSGL-1 to host defense against invasive pneumococcal disease. (A) Bacterial levels recovered at 6 h and 24 h from BALF, lung and blood of *PSGL-1* ^−/−^ mice (open bars) and wild-type mice (black bars) after pneumonia infection with the pneumococcal D39 strain. (B) Bacterial levels recovered from blood of *PSGL-1* ^−/−^ mice (open bars) and wild-type mice (black bars) after sepsis caused by D39 strain. (C) Survival rates of *PSGL-1* ^−/−^ mice (solid line) and wild-type mice (dotted line) after pneumococcal sepsis produced by D39 *S*. *pneumoniae* strain. (D) Bacterial levels recovered from blood of *PSGL-1* ^−/−^ mice (open bars) and wild-type mice (black bars) after intravenous infection with D39 strain. (E-F) Bacterial counts in blood and survival of *PSGL-1* ^−/−^ mice (open bars and solid line) and wild-type mice (black bars and dotted line) infected by the intraperitoneal route (sepsis) with TIGR4 strain. Error bars represent the SDs and asterisks indicate statistical significance of bacterial levels of *PSGL-1* ^−/−^ mice compared to wild-type mice. For differences in survival between wild-type mice and *PSGL1* ^−/−^ mice a long-rank test was used (*P* <0.01 for D39 strain and *P* <0.05 for TIGR4 strain).

The development of IPD depends of the complex interplay of many factors including virulence determinants of the pathogen and the efficacy of the host immune response to clear the infection process. A failure to efficiently detect and destroy *S*. *pneumoniae* in the lower respiratory tract or the systemic circulation lead to severe pneumonia or disseminated infection which are associated to increased mortality rates [3]. Next, the protective role of PSGL-1 against IPD was investigated using pneumonia and sepsis models of infection (Fig 5). In pneumonia, bacterial counts were slightly higher in the bronchoalveolar lavage fluid (BALF) and lung of *PSGL-1* ^−/−^ mice, and much higher in the blood of KO mice (Fig 5A). These bacterial levels were markedly elevated at 24 h in the blood of *PSGL-1* ^−/−^ mice (with the progression of the infection), confirming that PSGL-1 contributes to control bacterial load by reducing the severity of pneumococcal dissemination (Fig 5A). In the sepsis model, *PSGL-1* ^−/−^ mice had greater levels of bacteria in blood (Fig 5B). In addition, lethal infection developed faster in *PSGL-1* ^−/−^ mice than in wild-type mice indicating that PSGL-1 plays a critical role in host defense against IPD by controlling bacterial infection in the systemic circulation (Fig 5B and5C). To confirm this hypothesis and exclude the contribution of cellular migration mediated by PSGL-1, mice were directly infected by the intravenous route (Fig 5D). Mice lacking PSGL-1 had increased levels of bacteria in blood than wild-type mice both at 6 and 24 h confirming that a major function of PSGL-1 in host defense is to clear the bacteria in the bloodstream controlling the dissemination (Fig 5D). To extend the importance of PSGL-1 in the clearance of *S*. *pneumoniae* from the systemic infection, a sepsis model was repeated using a lethal dose of a different serotype such as TIGR4 strain. Our findings corroborated the results obtained with the D39 strain demonstrating that lack of PSGL-1 was associated with increased bacterial counts in blood and a more severe infection compared to wild-type mice (Fig 5E and 5F). Overall, our findings demonstrate that PSGL-1 plays an important role against IPD.

### PSGL-1 contributes to cellular migration and the inflammatory response

**Fig 6:**
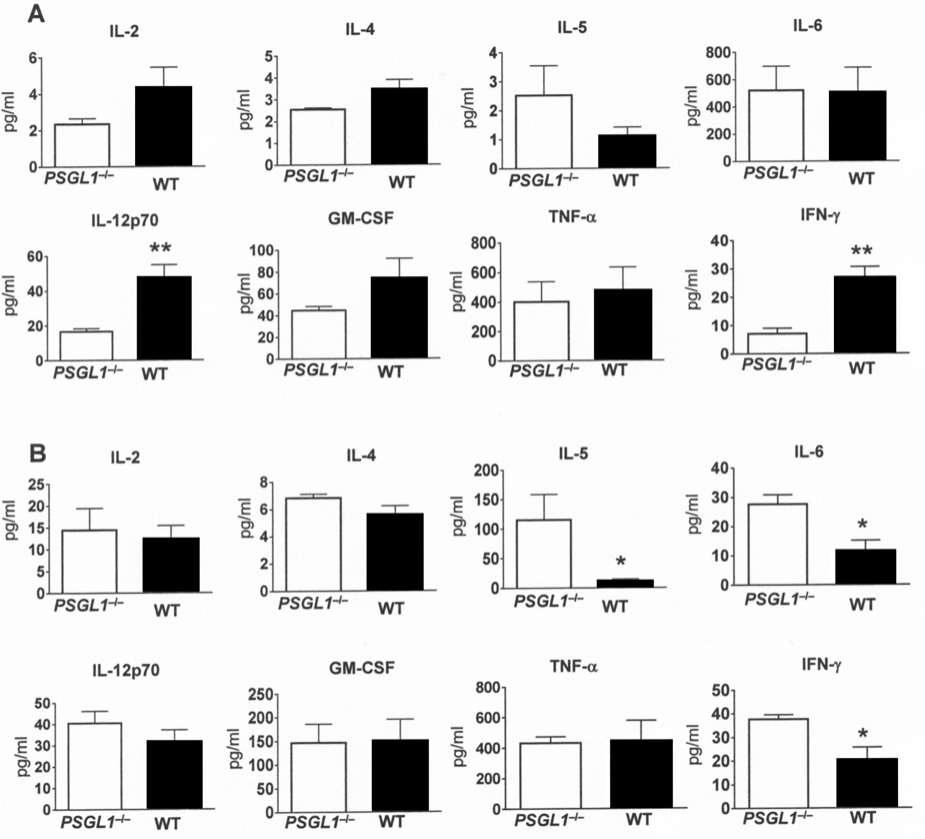
Impact of PSGL-1 deficiency in the inflammatory response after pneumococcal infection. Cytokine levels (pg/ml) in wild-type mice (black bars) and *PSGL-1* ^−/−^ mice (open bars) at 24 h after intranasal inoculation of *S*. *pneumoniae* D39 strain. (A) Cytokines determined in BALF. (B) Cytokines measured in sera. Error bars represent the SDs and asterisks indicate statistical significance of cytokine levels of *PSGL-1* ^−/−^ mice compared to wild-type mice.

The inflammatory response to infection with *S*. *pneumoniae* in *PSGL-1* ^−/−^ and wild-type mice was characterized in BALF and serum. The pattern of the major cytokines associated to infection was similar in BALF (Fig 6A), although in serum of *PSGL-1* ^−/−^ mice there were significant increased levels of IL-5, IL-6, and IFN-γ (*P* <0.05) which is compatible with the higher bacterial levels found in the blood of these mice (Fig 6B).

**Fig 7:**
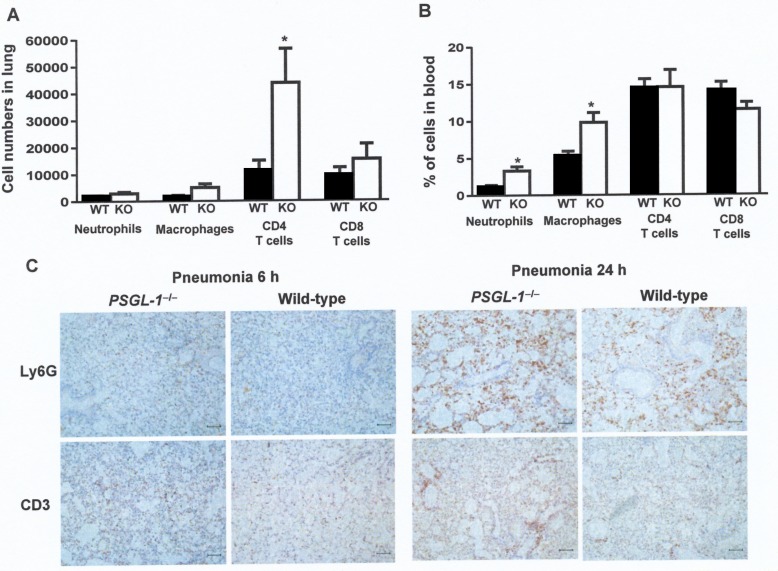
Contribution of PSGL-1 to cellular migration in lungs and blood of infected mice with *S*. *pneumoniae*. (A) Neutrophils, macrophages, CD4^+^T and CD8^+^T cells in the lungs of wild-type (black bars) or *PSGL-1* ^−/−^ mice (open bars) after pneumonia infection. (B) Proportion of neutrophils, macrophages, CD4^+^T and CD8^+^T cells in the blood of wild-type (black bars) or *PSGL-1* ^−/−^ mice (open bars) after intravenous infection. (C) Representative lung histological sections from infected mice that show granulocytes infiltrates in *PSGL-1* ^−/−^ mice visualized by Ly6G staining. T cell infiltrates were observed by CD3 staining. Original magnification × 10. Scale bars represent 200 μm.

Neutrophils predominate within cellular infiltrates in pneumococcal pneumonia and the consequences of the neutrophil influx for the host can be advantageous or detrimental, depending on the degree of cellular influx and the ability of the pathogen to successfully avoid the immune response [3]. Leukocyte infiltration into lungs and circulating leukocytes in blood, were measured by flow cytometry, using wild-type and *PSGL-1* ^−/−^mice infected with *S*. *pneumoniae* D39 strain (Fig 7A and 7B). In the pneumonia model (intranasal inoculation), the number of neutrophils and macrophages were similar in the lungs of both types of mice whereas T cell counts were higher in *PSGL-1* ^−/−^mice (Fig 7A). In a systemic model of infection (intravenous inoculation), however, the number of T cells were similar, although the proportion of neutrophils and macrophages were significantly higher in *PSGL-1* ^−/−^mice (Fig 7B), which is compatible with the higher levels of bacteria in the blood of these mice (Fig 5D). Overall, these results indicate that *PSGL-1* ^−/−^mice, despite having greater numbers of leukocytes in blood, had an impaired ability to clear the bacteria from the bloodstream confirming the importance of PSGL-1 in the recognition and killing of *S*. *pneumoniae* in the systemic circulation.

Immunohistochemical characterization of thin sections from lung tissues confirmed that mice deficient in PSGL-1 had greater infiltration of T cells and neutrophils compared to wild-type mice, which are consistent with the severity of the infection process developed in *PSGL-1* ^−/−^ mice (Fig 7C).

### LytA is relevant for the interaction with PSGL-1

**Fig 8:**
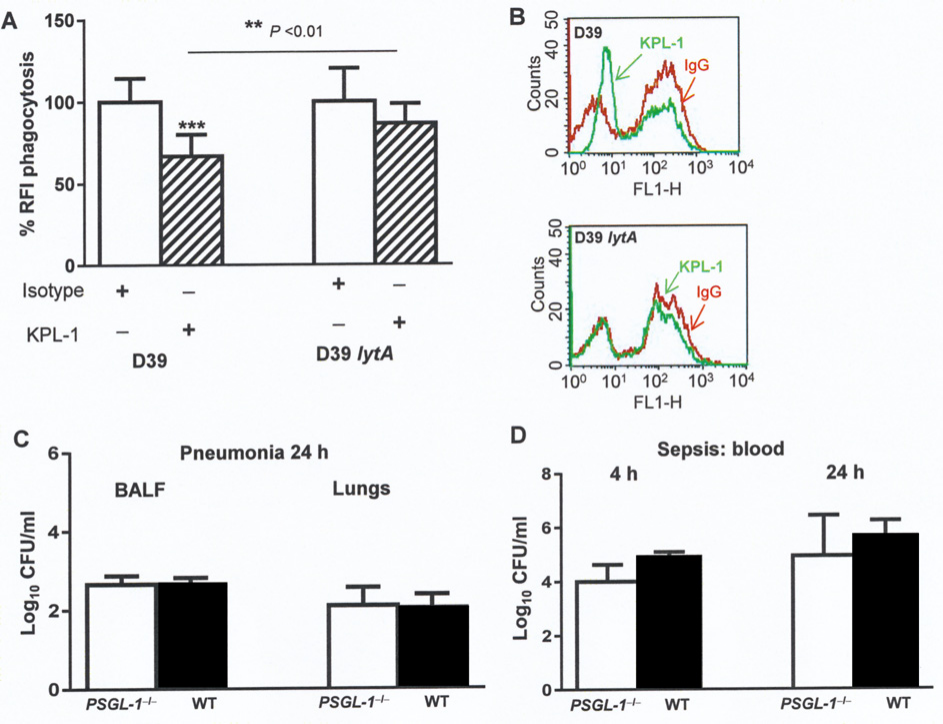
Lack of LytA counteracts PSGL-1 deficiency. (A) Phagocytosis of FAM-SE-labeled pneumococcal strains (D39, D39 *lytA*) using HL-60 cells exposed to IgG isotype control (open bar) or exposed to KPL-1 (striped bar). (B) Example of flow cytometry histograms showing phagocytosis by HL-60 cells exposed to IgG isotype control (red) or KPL-1 (green). Error bars represent the SDs and asterisks indicate statistical significance after incubation with KPL-1 compared to the exposure to IgG isotype control. **P <0.01 for the comparison between D39 *vs* D39 *lytA* in HL-60 cells exposed to KPL-1. (C) Bacterial levels recovered at 24 h from BALF and lungs of *PSGL-1* ^−/−^ or wild-type mice after pneumonia infection with the pneumococcal D39 *lytA* mutant. (D). Bacterial levels recovered at 4 h and 24 h from blood of *PSGL-1* ^−/−^ or wild-type mice after intraperitoneal infection with the pneumococcal D39 *lytA* strain.

To demonstrate that pneumococcal LytA is involved in the physiological effects mediated by PSGL-1, phagocytic assays were performed using HL-60 cells exposed or not to the KPL-1 antibody and a pneumococcal strain lacking LytA (Fig 8A and 8B). Hence, our findings confirmed that phagocytosis of a LytA deficient strain is not dependent on PSGL-1 on HL-60 cells (Fig 8A and 8B). To confirm the relevance of LytA in the interaction with PSGL-1, pneumonia and sepsis models of infection were repeated in wild-type and *PSGL-1* ^*−/−*^ mice using a D39 *lytA* deficient strain (Fig 8C and 8D). In contrast to mice infected with wild-type D39, there were no differences in lung or BALF CFU between *PSGL-1* ^−/−^ and wild-type mice infected intranasally with the D39 *lytA* mutant strain. Similarly, in the sepsis model there were no differences in recovered D39 *lytA* deficient strain CFU between *PSGL-1* ^−/−^ and wild-type mice. These results confirm that the interaction of LytA with PSGL-1 is important for innate immunity against *S*. *pneumoniae*. (Fig 8C and 8D).

## Discussion

Neutrophils are key players in the innate and adaptive immune responses to microbial cells, since they are critical for rapid clearance of invading bacteria [25, 26]. For this process, neutrophils must first detect the microorganisms using surface receptors that are essential to engulf and kill the pathogen [18, 27]. PSGL-1 is a ligand of P-, E- and L-selectins, and is able to mediate the tethering and rolling of circulating leukocytes on the activated endothelium prior to their extravasation [8, 11, 12]. The role of P, E and L-selectin as well as certain integrins against pneumococcal infection has been previously characterized [9, 10, 28, 29]. However, the direct role of PSGL-1 on leukocytes in host defense against *S*. *pneumoniae* including its contribution to the recognition and clearance of this microorganism is unknown. As this bacterium is highly variable with up to 96 serotypes described so far [30], we included different serotypes of *S*. *pneumoniae* to investigate the role of PSGL-1 in phagocytosis. Hence, our findings showed that PSGL-1 is involved in the recognition and phagocytosis of a major human pathogen such as *S*. *pneumoniae* contributing therefore to the variety of receptors on professional phagocytes that are needed to efficiently identify and destroy invading pathogens [4–6, 18]. For the detection of the pathogen it is necessary the interaction of phagocytic receptors with bacterial ligands that are usually exposed on the surface of the microorganism [19]. Using LytA-deficient mutants, we have recently demonstrated that this surface-exposed protein is a major determinant in the virulence of *S*. *pneumoniae* by interacting with essential components of the immune system including phagocytes [20]. Our results show now that the LytA autolysin is recognized by PSGL-1 and this effect is dependent on the level of PSGL-1 suggesting that variations in the expression of PSGL-1 on leukocytes might contribute to the efficiency of this interaction. One of the major concerns in the outcome of the infection is that pneumococcal disease can be produced by clinical isolates of a wide variety of polysaccharide capsules, the main virulence determinant of *S*. *pneumoniae* [22, 23]. In this sense, using pneumococcal transformants expressing the same genetic background but different CPS, we have demonstrated that PSGL-1 recognizes the capsule of *S*. *pneumoniae*. This effect was variable depending on the CPS expressed, with the lowest binding related to the strain expressing serotype 19A. In this sense, incidence of IPD cases caused by this serotype has dramatically increased in the last few years and it has been linked to the emergence of vaccine escape variants that arise by switching the capsular locus from serotype 4 to 19A [31, 32]. These results are important in terms of pathogenesis because differences in the recognition of pneumococcal CPS by receptors such as PSGL-1, might explain why certain serotypes of *S*. *pneumoniae* are more associated to IPD and dissemination worldwide than others [31–33].

The repertoire of host receptors involved in the binding, uptake, signaling and response to invading pathogens is critical for the outcome of the infection. PSGL-1 is the main selectin receptor involved in neutrophil adhesion and migration [8]. Although the importance of leukocyte extravasation is relevant in pneumococcal infection [9, 10, 29], the contribution of PSGL-1 to the resolution of IPD was previously unknown. In this study we have used *PSGL-1* ^−/−^mice to investigate the in vivo role of this receptor in host defense against pneumococcal infection. Our findings confirm that PSGL-1 may act as a pathogen-recognition receptor of the immune system [34]. In this sense, PSGL-1 acts in host defense controlling bacterial proliferation, dissemination and tissue injury which are critical aspects of IPD. This is of great relevance from the respiratory perspective as invasive pneumonia caused by *S*. *pneumoniae* is one of the major causes of mortality in children and adults [1, 3]. Lack of PSGL-1 has been linked to a greater susceptibility against the intracellular pathogens *Salmonella typhimurium* or *Citrobacter rodentium*, which is associated with dramatically increased levels of pro-inflammatory cytokines after intestinal infection [35, 36]. In the case of *S*. *pneumoniae*, bacterial recognition by immune cells generates an array of cytokines which may play a significant role in host defense. Increase of IL-5, IL-6 and IFN-γ was observed in *PSGL-1* ^–/–^mice sera. According to our findings, it has been described that increased serum levels of IL-5 and IL-6 were associated with reduced microbial clearance and higher mortality rates in sepsis [37, 38]. In addition, the increased levels of IL-12 and IFN-γ in the lungs of wild-type mice might be protective as IFN-γ is involved in the IL-12 regulation of neutrophil-mediated host defense against pneumococcal pneumonia [39].

Bacterial infections of the lower respiratory tract are characterized by massive accumulation of neutrophils in the alveolar spaces [3, 40]. Our results show that similar numbers of neutrophils and macrophages were observed in the lungs of *PSGL-1* ^−/−^ mice. Our results might be unexpected as PSGL-1 is involved in cellular migration of these cells and therefore, increased numbers should be present in the lungs of wild-type mice [8]. Hence, our findings can be explained in the context of a bacterial infection as certain pathogens ―including *S*. *pneumoniae*― have the ability to impair neutrophil migration to the site of infection by cleaving PSGL-1 [41, 42]. Interestingly, this is in line with previous findings confirming that the absence of endothelial selectins E, L and P is not associated with impairment of leukocyte emigration to infection sites after pneumococcal disease [10, 43]. In addition, PSGL-1 negatively regulates CD4^+^T cell immune responses *in vivo* which can explain the increased levels of T cells observed in the lungs of *PSGL-1* ^−/−^ infected mice [44].

Using an intravenous infection model when no collateral migration effects were expected, higher numbers of neutrophils and macrophages but not T cells, were observed in *PSGL-1* ^−/−^ mice. This is consistent with previous observations showing that blood of *PSGL-1* ^−/−^ mice contained similar numbers of lymphocytes although they had a significant increase in the proportion of leukocytes, with enhanced levels of granulocytes and monocytes in comparison to wild-type mice [45]. This is relevant from the phagocytosis perspective as *PSGL-1* ^−/−^ mice, despite having increased numbers of these phagocytic cells in the bloodstream, were unable to control bacterial replication in the blood, leading to the rapid development of fatal infection. Overall, these results confirm that PSGL-1 on leukocytes plays a critical role in host defense against pneumococcal infection. As a consequence of pathogen-recognition by PSGL-1, *S*. *pneumoniae* is efficiently engulfed and destroyed, reducing bacterial replication and dissemination in the host, contributing to control the severity of IPD.

## Materials and Methods

### Ethics statement

All the experiments involving the use of animals have been performed following the guidelines of the Bioethical and Animal Welfare Committee of Instituto de Salud Carlos III (ISCIII) that reviewed and approved protocol CBA PA 52-2011-v2, to be performed at the National Centre for Microbiology of ISCIII. Animals were bred at Universidad Autónoma de Madrid animal facility following institutional guidelines for animal use and care. Infection experiments conformed to the Spanish government legislation (RD 1201/2005) and European Community regulations (86/609/EEC).

### Bacterial strains and growth conditions

The *S*. *pneumoniae* strains used were D39 (NCTC 07466, serotype 2), TIGR4 (ATCC BAA-334, serotype 4), and clinical isolates of different serotypes obtained from the Spanish Pneumococcal Reference Laboratory; 957/12 (serotype 3), 1515/97 (serotype 6B), 450/12 (serotype 11A), 3347/12 (serotype 19A), 69 (serotype 19F) and 48 (serotype 23F). The non-encapsulated *S*. *pneumoniae* strain M11 and their isogenic transformants expressing CPS of serotypes 3, 6B and 19A were also included in this study [46]. *S*. *pneumoniae* D39 strain expressing the GFP was constructed by transformation with plasmid pMV158GFP [47] and used for confocal microscopy experiments. *S*. *pneumoniae* strains were cultured on blood agar plates at 37°C in a 5% CO_2_ atmosphere, or in Todd-Hewitt broth supplemented with 0.5% yeast extract, to an optical density at 550 nm (OD_550_) of 0.5, and stored at −70°C in 10% glycerol as single use aliquots.

### Phagocytosis by HL-60 cells and neutrophils isolated from mice

HL-60 cells (CCL-240; ATCC) differentiated to granulocytes were used and the general conditions of the assay were based on those described previously [17, 48]. Briefly, *S*. *pneumoniae* strains were fluorescently labeled by incubation with FAM-SE (Molecular Probes) in 0.1 M sodium bicarbonate buffer for 1 h at 37°C, washed five times with Hanks balanced salt solution (HBSS) and stored at −70°C in 10% glycerol as aliquots for further assays. HL-60 cells were harvested by centrifugation and washed twice with HBSS and once with HBSS in the presence of calcium and magnesium ions. Infection assays were performed in the absence of serum to avoid complement-dependent phagocytosis and 10^5^ HL-60 cells were infected with 2×10^6^ colony forming units (CFU) of viable FAM-SE labeled bacteria. To block PSGL-1, HL-60 cells were incubated for 1 h at room temperature with 25 μg/ml of the KPL-1 antibody (mouse anti-human PSGL-1; MBL) or IgG isotype control (mouse anti-human IgG; Novus Biologicals) as previously described [15, 16]. A similar approach was performed using purified LytA. A minimum of 6,000 cells were analyzed using a FACS Calibur flow cytometer (BD Biosciencies). Using cytochalasin D, an inhibitor of actin polymerization, we have previously shown that the majority of the effect on the association of fluorescent *S*. *pneumoniae* with HL-60 cells is due to phagocytosis rather than to adhesion of bacteria to the cell surface [49]. Results were expressed as a fluorescence index defined as the proportion of positive cells for fluorescent bacteria multiplied by the geometric mean of fluorescence intensity which correlates with the amount of bacteria phagocytosed per cell [48].

Opsonophagocytosis killing assays were performed in the absence of serum using 10^5^ HL-60 cells with the PSGL-1 receptor either active or blocked as mentioned above and 2.5 × 10^4^ CFU/ml of *S*. *pneumoniae* as previously described [50]. Serial dilutions of culture supernatants were plated on blood agar plates for bacterial counts determination and results were expressed as bacterial survival after 45 min incubation of the pneumococcal strains with HL-60 cells expressing (or not) PSGL-1.

Phagocytosis assays were repeated using neutrophils purified from the spleen of wild-type and *PSGL-1* ^−/−^ mice by FACS using a FACSAria I (BD Biosciences) dispositive with DIVA version 6.1 software as previously described [51]. Briefly, single-cell suspensions were prepared in staining buffer (2% fetal calf serum in PBS), and non-specific binding was blocked with Fc block (BD Biosciences). Staining was performed using standard protocols with the following antibodies diluted 1/200 in staining buffer including propidium iodide (rat anti-mouse CD11b-allophycocyanin (APC) and rat anti-mouse GR-1-phycoerythrin; Biolegend).

### Confocal microscopy

*S*. *pneumoniae* strains labeled with FAM-SE were used for immunofluorescence microscopy. HL-60 cells previously infected as described above were seeded on 12-mm circular coverslips for immunofluorescence staining. As HL-60 cells are in suspension, cells were cytofuged at 70 × *g* for 2 min using a Cytospin centrifuge (Thermo Electron, Pittsburgh, PA), as described elsewhere [16]. For the detection of PSGL-1 in HL-60 cells differentiated to granulocytes, cells were fixed with 3% paraformaldehyde (PFA) for 10 min at room temperature and after two washes with PBS, coverlips were kept in a solution 1 M NH_4_Cl-PBS solution. Coverslips containing the infected cells were washed twice in PBS containing 0.1% saponin and once in PBS and incubated for 30 min with the primary antibody. Staining was performed in PBS containing 10% horse serum, 0.1% saponin and the primary antibody using a mouse anti-human PSGL-1 monoclonal antibody (KPL-1; MBL) diluted 1/300. Cellular DNA was stained with Hoechst (Invitrogen) diluted 1/2500. After 30 min incubation with the primary antibody at room temperature, coverlips were washed twice with PBS-saponin 0.1%, and once with PBS pH 7.0 before incubation during 30 min at room temperature with a dilution 1/200 of the secondary antibody (goat anti-mouse Texas Red; Serotec). Finally, coverslips were washed twice in PBS containing 0.1% saponin, once in PBS, and once in H_2_O, mounted with Aqua Poly/Mount (Polysciences), and analyzed with a Leica spectral SP5 confocal microscope using the Leica software (LAS-AF).

### PSGL-1 binding to *S*. *pneumoniae*

Binding of PSGL-1 to *S*. *pneumoniae*, purified LytA or CPS was analyzed by ELISA as previously described [20]. Briefly, whole cell ELISA was performed by coating 96-well plates with 200 μl of exponentially growing bacteria and resuspended in PBS to an OD_550_ of 1.0. Plates were air dried at room temperature and blocked with 200 μl of PBS-0.5% BSA-NaN_3_ for 1 h before 50 μl of different concentrations of PSGL-1 (R&D systems, USA) were added to each well. After overnight incubation at 4°C, plates were washed 5 times with PBS-Tween 0.1% and incubated overnight at 4°C with 50 μl of mouse anti-human PSGL-1 (KPL-1; MBL) diluted 1/4000. After 5 washes with PBS-Tween 0.1%, plates were incubated with goat anti-mouse IgG HRP (Southern Biotech) for 30 min at room temperature and developed with *o*-phenylenediamine (Sigma-Aldrich). Plates were measured at OD_492_ using a microtiter plate reader (Anthos 2020). Direct binding of PSGL-1 to purified LytA protein or type 3 CPS (ATCC 169-X, Merck Sharp & Dohme) was performed as described above except that the 96-well plates were coated with 50 μg of purified LytA protein or CPS per well. Purified LytA protein was obtained by overexpression in *Escherichia coli* [52].

### Experimental models of infection

Wild-type C57BL/6 mice and *PSGL-1* ^−/−^ mice were bred in a conventional animal facility at the School of Medicine, Universidad Autónoma de Madrid (UAM). *PSGL-1* ^−/−^ mice were kindly provided by Dr. D Vestweber and Dr. MK Wild (Max Plank Institute for Molecular Biomedicine, Münster, Germany). Wild-type C57BL/6 mice obtained from the Jackson Laboratory and *PSGL-1* ^−/−^ mice were backcrossed and the wild-type and *PSGL-1* ^−/−^ littermates obtained from crosses of the resulting heterozygous mice were used to breed our wild-type and *PSGL-1* ^−/−^ colonies used in this study. Animal procedures were approved by the Animal Care and Use Committee of ISCIII. All mice used were 8–16 weeks old, and within each experiment, groups of mice were matched for age and sex. Studies investigating pneumococcal sepsis or pneumonia were performed using groups of at least 5 mice and infected as previously described [48]. Briefly, for sepsis, mice were challenged with 5 × 10^6^ CFU/ml for D39 strain or 3 × 10^4^ CFU/ml for TIGR4 strain (in a volume of 200 µl) by the intraperitoneal route, whereas for pneumonia, mice under anesthesia with isofluorane were inoculated intranasally with 50 µl containing 10^7^ CFU/mouse of D39 strain. For intravenous inoculation, mice were infected with 2 × 10^7^ CFU/mouse of D39 strain through the tail vein. At 6 h and 24 h after challenge, a lethal dose of pentobarbital was administered and bacterial counts were determined from samples recovered from BALF, lung and blood. Experiments were repeated twice using 5 mice in each group and results were expressed as Log_10_ CFU/ml of bacteria recovered from the different sites. Cytokines were measured from BALF and blood of wild-type mice and *PSGL-1* ^−/−^ mice infected with D39 strain by the intranasal route as explained above. Cytokines levels (IL-2, IL-4, IL-5, IL-6, IL-10, IL-12p70, GM-CSF, TNF-α and IFN-γ) were determined by using a Luminex magnetic bead array assay (Bio-Rad) according to manufacturer protocols.

Experiments investigating cellular populations in lungs and blood after pneumococcal infection were performed in wild-type and *PSGL-1* ^−/−^ mice infected as explained above using a FACS assay as previously described [51, 53]. Briefly, single-cell suspensions were prepared in staining buffer (2% fetal calf serum in PBS), and non-specific binding was blocked with Fc block (BD Biosciences). Staining was performed using standard protocols with the following antibodies diluted 1/400 in staining buffer (rat anti-mouse CD11b-allophycocyanin (APC), Biolegend; rat anti-mouse CD4-FITC, Biolegend; rat anti-mouse CD8-APC, Biolegend; rat anti-mouse GR-1-phycoerythrin; Biolegend) [51, 53]. Cells were analyzed on a FACSCanto I using the FlowJo version 6.3.4 software package.

### Lung sectioning and histology

Mice were euthanized with pentobarbital and lungs were inflated and fixed with 4% PFA in PBS. Lungs were paraffin-embedded and 5-μm sections were obtained. Infiltrates of granulocytes and T cells were measured by staining with anti-Ly-6G/6C (antigen retrieval pH 6.5, 1/400, Abcam ab2557) and anti-CD3 (antigen retrieval pH 6.0, 1/200, Santa Cruz Biotechnology sc-1127) antibodies respectively. Immunohistochemistry was performed with the Dako LSAB^+^ System-HRP following manufacturer’s instructions.

### Statistical analysis

Data are representative of results obtained from repeated independent experiments, and each data point represents the mean and standard deviations (SD) for 3 to 5 replicates. Statistical analysis was performed by using two-tailed Student’s *t* test (for two groups), whereas analysis of variance (ANOVA) followed by a Dunnett’s *post hoc* test was chosen for multiple comparisons. Survival was analyzed by the log-rank test. GraphPad InStat version 5.0 (GraphPad Software, San Diego, CA) was used for statistical analysis. Differences were considered statistically significant with *P* <0.05 (*) and highly significant with *P* <0.01 (**) and *P* <0.001 (***).

## Supporting Information

S1 Fig Expression of receptors on HL-60 cells and impact of FCγ-receptors in the phagocytosis mediated by PSGL-1. (A) Expression of different receptors on HL-60 cells exposed or not to dimethylformamide (DMF) for granulocytic differentiation. (B) Phagocytosis of FAM-SE-labeled D39 strain using HL-60 cells exposed to KPL-1 (open bar) or exposed to KPL-1 and Fcγ-R blocking agent (striped bar) in combination.
(TIF)

S2 Fig Co-localization of PSGL-1 and *S*. *pneumoniae*. Local co-localization of FAM-SE *S*. *pneumoniae* (green), Cellular DNA (blue) and PSGL-1 (red) is shown and quantified by plotting the fluorescence intensity and the distance (in μm).
(TIF)

S3 Fig Previous exposure of HL-60 cells to LytA impairs the uptake. (A) Blockage of PSGL-1-mediated phagocytosis by preincubation with purified LytA. HL-60 cells were incubated for 1 h with 0.1μg/ml or 0.01 μg/ml of purified LytA (open bars) or without LytA (black bar) before infection with D39 strain. (B) Example of flow cytometry histogram of cells incubated with (red) or without (green) LytA. Error bars represent the SDs and asterisks indicate statistical significance of pneumococcal phagocytosis by HL-60 cells preincubated with purified LytA in comparison to incubation without LytA.
(TIF)

S1 Movie Recognition and phagocytosis of *S*. *pneumoniae* is enhanced when PSGL-1 is fully functional. HL-60 cells with the PSGL-1 active were infected for 30 min with the D39 strain expressing GFP and visualized by live imaging confocal microscopy and environmental control. Bacteria are markedly recognized and trapped by the cells.
(MPG)

S2 Movie Recognition and phagocytosis of *S*. *pneumoniae* is impaired when PSGL-1 is blocked. HL-60 cells with the PSGL-1 blocked by KPL-1 treatment for 1h were infected for 30 min with the D39 strain expressing GFP and visualized by live imaging confocal microscopy and environmental control. Bacterial uptake is impaired and *S*. *pneumoniae* diverts the phagocytosis process very efficiently.
(MPG)
