# Supplementary figures and images for: PSGL-1 on Leukocytes is a Critical Component of the Host Immune Response against Invasive Pneumococcal Disease
Source: PLoS Pathog. 2016 Mar 14;12(3):e1005500. doi: 10.1371/journal.ppat.1005500 (PMC4790886; doi:10.1371/journal.ppat.1005500)

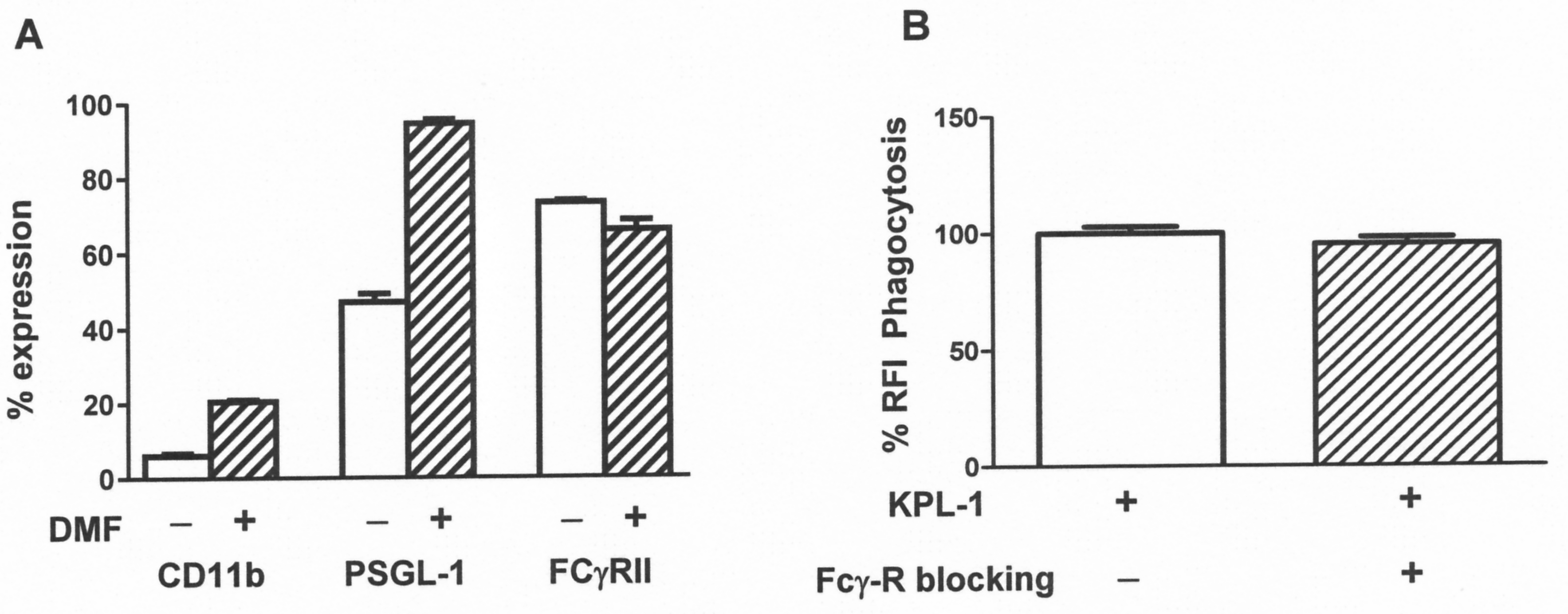

Supplement: S1 Fig — (A) Expression of different receptors on HL-60 cells exposed or not to dimethylformamide (DMF) for granulocytic differentiation. (B) Phagocytosis of FAM-SE-labeled D39 strain using HL-60 cells exposed to KPL-1 (open bar) or exposed to KPL-1 and Fcγ-R blocking agent (striped bar) in combination. (TIF) [file ppat.1005500.s001.tif]

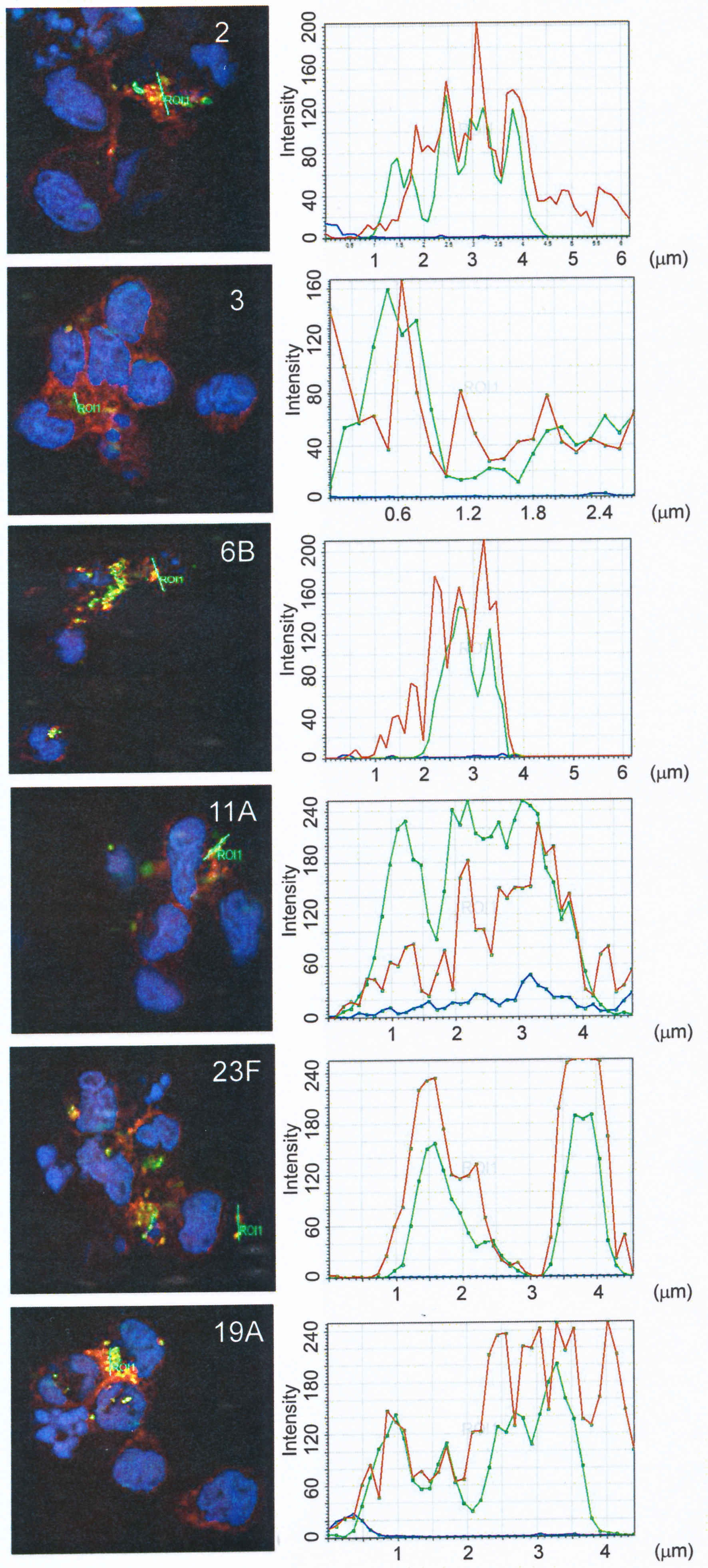

Supplement: S2 Fig — Local co-localization of FAM-SE S. pneumoniae (green), Cellular DNA (blue) and PSGL-1 (red) is shown and quantified by plotting the fluorescence intensity and the distance (in μm). (TIF) [file ppat.1005500.s002.tif]

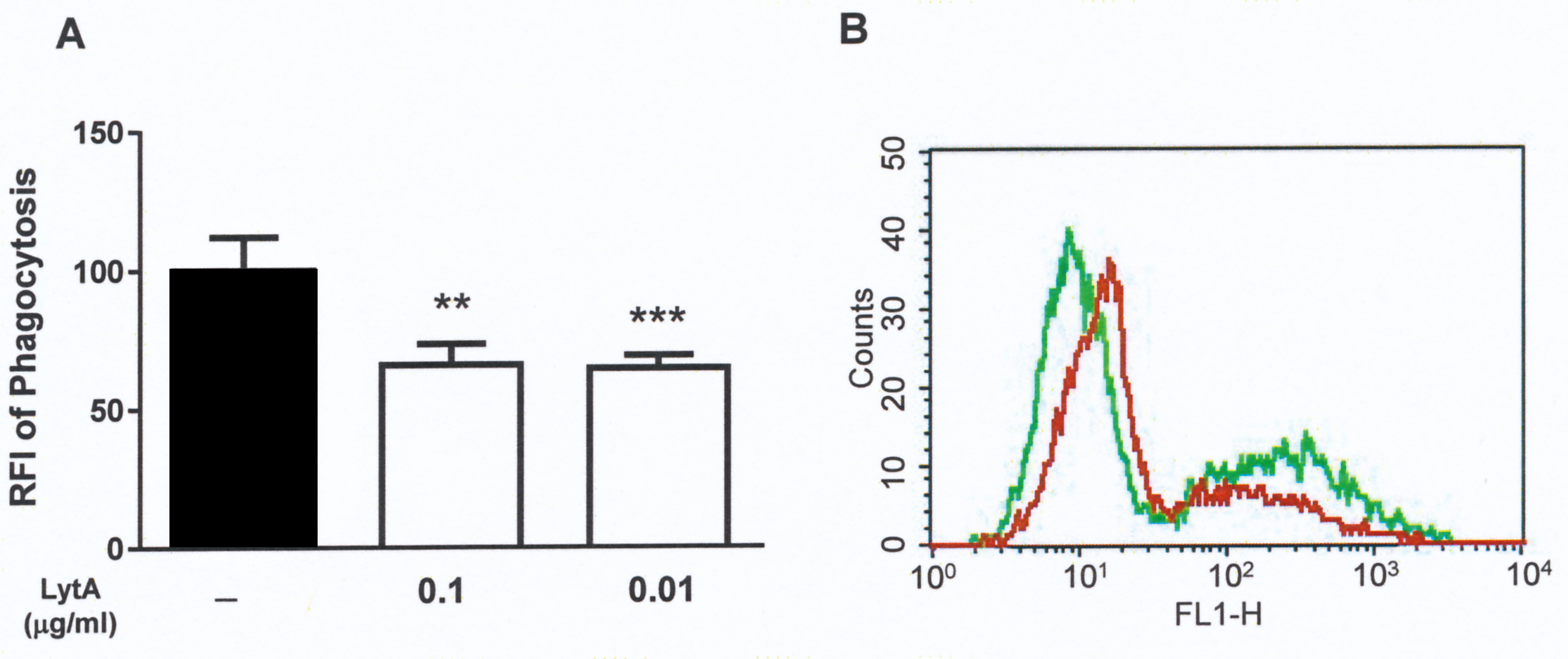

Supplement: S3 Fig — (A) Blockage of PSGL-1-mediated phagocytosis by preincubation with purified LytA. HL-60 cells were incubated for 1 h with 0.1μg/ml or 0.01 μg/ml of purified LytA (open bars) or without LytA (black bar) before infection with D39 strain. (B) Example of flow cytometry histogram of cells incubated with (red) or without (green) LytA. Error bars represent the SDs and asterisks indicate statistical significance of pneumococcal phagocytosis by HL-60 cells preincubated with purified LytA in comparison to incubation without LytA. (TIF) [file ppat.1005500.s003.tif]
